# Bis(1,4-diazo­niabi­cyclo­[2.2.2]octa­ne) di-μ-chlorido-bis­[tetra­chlorido­anti­monate(III)] dihydrate

**DOI:** 10.1107/S160053681301307X

**Published:** 2013-05-18

**Authors:** Tarek Ben Rhaiem, Habib Boughzala, Ahmed Driss

**Affiliations:** a Laboratoire de Matériaux et Cristallochimie, Faculté des Sciences de Tunis, Université de Tunis El Manar, 2092 Manar II Tunis, Tunisia

## Abstract

The title salt, (C_6_H_14_N_2_)_2_[Sb_2_Cl_10_]·2H_2_O, was obtained by slow evaporation of an acidic solution of 1,4-di­aza­bicyclo­[2.2.2]octane and SbCl_3_. The crystal structure consists of (C_6_H_14_N_2_)^2+^ cations, [Sb_2_Cl_10_]^4−^ double octa­hedra and lattice water mol­ecules. All mol­ecular components are situated on special positions. The cation and the lattice water mol­ecule exhibit mirror symmetry, whereas the anion has site symmetry 2/*m*. The cations, anions and water mol­ecules are alternately arranged into columns along [010]. Individual columns are joined into layers extending along (001). Intra­layer N—H⋯O and inter­layer N—H⋯Cl hydrogen-bonding inter­actions lead to the formation of a three-dimensional network.

## Related literature

For background to this class of compounds, see: Pietraszko *et al.* (2001 ▶); Feng *et al.* (2007 ▶); Bujak & Zaleski (1999 ▶); Knodler *et al.* (1988 ▶); Baker & Williams (1978 ▶). For a related structure, see: Qu & Sun (2005 ▶).
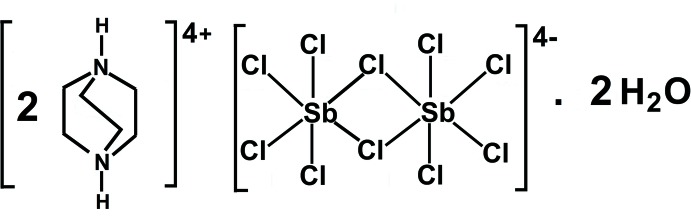


## Experimental

### 

#### Crystal data

(C_6_H_14_N_2_)_2_[Sb_2_Cl_10_]·2H_2_O
*M* *_r_* = 862.46
Orthorhombic, 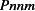

*a* = 9.162 (1) Å
*b* = 20.869 (7) Å
*c* = 7.566 (2) Å
*V* = 1446.8 (7) Å^3^
*Z* = 2
Mo *K*α radiation
μ = 2.81 mm^−1^
*T* = 298 K
0.50 × 0.43 × 0.36 mm

#### Data collection

Enraf–Nonius CAD-4 diffractometer
Absorption correction: ψ scan (North *et al.*, 1968 ▶) *T*_min_ = 0.334, *T*_max_ = 0.431
2797 measured reflections
1700 independent reflections
1488 reflections with *I* > 2σ(*I*)
*R*_int_ = 0.029
2 standard reflections every 120 min intensity decay: 1%

#### Refinement

*R*[*F* ^2^ > 2σ(*F* ^2^)] = 0.023
*wR*(*F* ^2^) = 0.061
*S* = 1.08
1700 reflections
82 parameters
H-atom parameters constrained
Δρ_max_ = 0.59 e Å^−3^
Δρ_min_ = −0.37 e Å^−3^

### 

Data collection: *CAD-4 EXPRESS* (Duisenberg, 1992 ▶); cell refinement: *CAD-4 EXPRESS*; data reduction: *XCAD4* (Harms & Wocadlo, 1995 ▶); program(s) used to solve structure: *SHELXS97* (Sheldrick, 2008 ▶); program(s) used to refine structure: *SHELXL97* (Sheldrick, 2008 ▶); molecular graphics: *DIAMOND* (Brandenburg, 2006 ▶); software used to prepare material for publication: *publCIF* (Westrip, 2010 ▶).

## Supplementary Material

Crystal structure: contains datablock(s) I, global. DOI: 10.1107/S160053681301307X/wm2740sup1.cif

Structure factors: contains datablock(s) I. DOI: 10.1107/S160053681301307X/wm2740Isup2.hkl

Additional supplementary materials:  crystallographic information; 3D view; checkCIF report

## Figures and Tables

**Table 1:** Hydrogen-bond geometry (Å, °)

*D*—H⋯*A*	*D*—H	H⋯*A*	*D*⋯*A*	*D*—H⋯*A*
N1—H1⋯Cl2	0.91	2.59	3.340 (4)	140
N1—H1⋯Cl3	0.91	2.77	3.390 (3)	126
N1—H1⋯Cl3^i^	0.91	2.77	3.390 (3)	126
N2—H2⋯O	0.91	2.00	2.780 (4)	143

Symmetry code: (i) 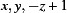.

**Fig. 1.**
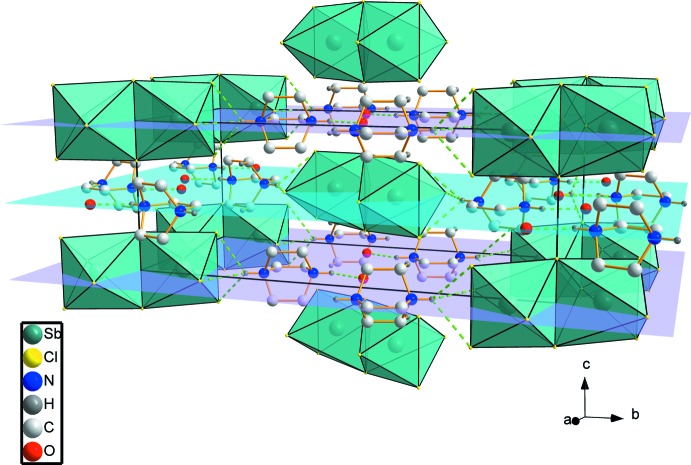
Three-dimensional view of (C6H14N2)2[Sb2Cl10].2H2O showing the crystal packing. H atoms not involved in hydrogen bonding (dashed lines) have been omitted for clarity.

**Fig. 2.**
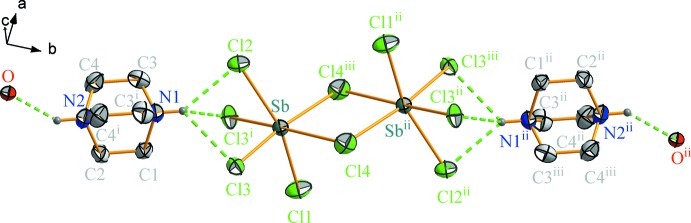
An ORTEP plot of the molecular entities of (C6H14N2)2[Sb2Cl10].2H2O, showing the atom numbering scheme. Displacement ellipsoids are drawn at the 50% probability level and H atoms are shown as small spheres of arbitrary radius. [Symmetry code: (i) x, y, 1 - z; (ii) 1 - x, 1 - y, z; (iii) 1 - x, 1 - y, 1 - z.]

## supplementary crystallographic information

### Comment

Halogenidoantimonates(III) and halogenidobismuthates(III) with organic cations defined by the general formula *R*_a_*M*_b_*X*_3b+a_ (where *R* is an organic cation; *M* is Sb^III^ or/and Bi^III^ and *X* is Cl, Br or/and I) are an interesting group of compounds due to their ferroelectric properties (Pietraszko *et al.*, 2001). Halogenidoantimonates(III) constitute a group of salts in which a number of compounds have a similar structural arrangement (Feng *et al.*, 2007; Bujak & Zaleski, 1999; Knodler *et al.*, 1988; Baker & Williams, 1978). Recently, the new chloridoantimonate(III), (C_6_H_14_N_2_)_2_[Sb_2_Cl_10_]^.^2H_2_O, has been synthesized in our laboratory. The synthesis and the structure determination are presented here.

The crystal structure of the title compound is formed by an alterning packing of layers along [001] (Fig. 1). Each layer spreads parallel to (001) and is located at *x* = 0 and *x* = 0.5 and consists of columns extending along [010] of alternating cations, anions and water molecules (Fig. 2).

The [Sb_2_Cl_10_]^4-^ anion has site symmetry 2/*m* and is composed of two distorted, edge-sharing SbCl_6_ octahedra. The *cis* Sb–Cl–Sb angles vary from 81.11 (2) to 104.42 (1)°, whereas the *trans* angles are between 164.64 (4) and 173.84 (1)°. The longest Sb1–Cl4 bond length (2.9107 (8) Å) corresponds to the bridging chlorine atom while the shortest one, Sb1–Cl3 (2.4904 (7) Å) is terminal and located in opposite direction to the bridging one (Fig. 2). The anionic charge is balanced by organic (C_6_H_14_N_2_)^2+^ (DABCO) cations that exhibit mirror symmetry. Bond lengths and angles in the (C_6_H_14_N_2_)^2+^ cation are within normal ranges and are comparable with those observed in a related structure (Qu & Sun, 2005).

The cohesion of the layers is ensured by N—H···O and N—H···Cl hydrogen bonds between organic cations, inorganic anions and the water molecules (Fig. 2, Table 1).

### Experimental

A mixture of SbCl_3_ (0.23 g, 1 mmol) and DABCO (0.11 g, 1 mmol) was dissolved in an aqueous solution of hydrochloric and stirred for several minutes at room temperature. Colorless crystals suitable for X-ray diffraction analysis were obtained by slow evaporation at room temperature over 2 weeks.

### Refinement

Hydrogen positions of the water molecule could not be located reliably and were eventually omitted from refinement. The C—H and N—H hydrogen atom positions were placed geometrically. They were included in the refinement using the riding-model approximation, with distance constraints of C—H = 0.97 Å, N—H = 0.91 and with *U*_iso_(H) = 1.2*U*_eq_(C,N).

### Crystal data

**Table:**

(C_6_H_14_N_2_)_2_[Sb_2_Cl_10_]·2H_2_O	*D*_x_ = 1.980 Mg m^−^^3^
*M**_r_* = 862.46	Melting point: 571 K
Orthorhombic, *P**n**n**m*	Mo *K*α radiation, λ = 0.71073 Å
Hall symbol: -P 2 2n	Cell parameters from 1700 reflections
*a* = 9.162 (1) Å	θ = 2.4–27.0°
*b* = 20.869 (7) Å	µ = 2.81 mm^−^^1^
*c* = 7.566 (2) Å	*T* = 298 K
*V* = 1446.8 (7) Å^3^	Prism, colourless
*Z* = 2	0.50 × 0.43 × 0.36 mm
*F*(000) = 840	

### Data collection

**Table:**

Enraf–Nonius CAD-4 diffractometer	1488 reflections with *I* > 2σ(*I*)
Radiation source: fine-focus sealed tube	*R*_int_ = 0.029
Graphite monochromator	θ_max_ = 27.0°, θ_min_ = 2.4°
non–profiled ω/2θ scans	*h* = −1→11
Absorption correction: ψ scan (North *et al.*, 1968)	*k* = −26→1
*T*_min_ = 0.334, *T*_max_ = 0.431	*l* = −9→3
2797 measured reflections	2 standard reflections every 120 min
1700 independent reflections	intensity decay: 1%

### Refinement

**Table:**

Refinement on *F*^2^	Secondary atom site location: difference Fourier map
Least-squares matrix: full	Hydrogen site location: inferred from neighbouring sites
*R*[*F*^2^ > 2σ(*F*^2^)] = 0.023	H-atom parameters constrained
*wR*(*F*^2^) = 0.061	*w* = 1/[σ^2^(*F*_o_^2^) + (0.0268*P*)^2^ + 1.1425*P*] where *P* = (*F*_o_^2^ + 2*F*_c_^2^)/3
*S* = 1.08	(Δ/σ)_max_ = 0.001
1700 reflections	Δρ_max_ = 0.59 e Å^−^^3^
82 parameters	Δρ_min_ = −0.37 e Å^−^^3^
0 restraints	Extinction correction: *SHELXL97* (Sheldrick, 2008), Fc^*^=kFc[1+0.001xFc^2^λ^3^/sin(2θ)]^-1/4^
Primary atom site location: structure-invariant direct methods	Extinction coefficient: 0.0099 (5)

### Special details

**Table:**

Experimental. Number of psi-scan sets used was 5 Theta correction was applied. Averaged transmission function was used. No Fourier smoothing was applied.
Geometry. All e.s.d.'s (except the e.s.d. in the dihedral angle between two l.s. planes) are estimated using the full covariance matrix. The cell e.s.d.'s are taken into account individually in the estimation of e.s.d.'s in distances, angles and torsion angles; correlations between e.s.d.'s in cell parameters are only used when they are defined by crystal symmetry. An approximate (isotropic) treatment of cell e.s.d.'s is used for estimating e.s.d.'s involving l.s. planes.
Refinement. Refinement of *F*^2^ against ALL reflections. The weighted *R*-factor *wR* and goodness of fit *S* are based on *F*^2^, conventional *R*-factors *R* are based on *F*, with *F* set to zero for negative *F*^2^. The threshold expression of *F*^2^ > σ(*F*^2^) is used only for calculating *R*-factors(gt) *etc*. and is not relevant to the choice of reflections for refinement. *R*-factors based on *F*^2^ are statistically about twice as large as those based on *F*, and *R*- factors based on ALL data will be even larger.

### Fractional atomic coordinates and isotropic or equivalent isotropic displacement parameters (Å^2^)

**Table:**

	*x*	*y*	*z*	*U*_iso_*/*U*_eq_	Occ. (<1)
Sb	0.40193 (3)	0.407356 (11)	0.5000	0.03327 (11)	
Cl1	0.16885 (14)	0.46510 (7)	0.5000	0.0678 (4)	
Cl2	0.62462 (11)	0.31027 (6)	0.5000	0.0471 (3)	
Cl3	0.30372 (8)	0.33776 (3)	0.26053 (10)	0.04669 (19)	
Cl4	0.5000	0.5000	0.23812 (14)	0.0534 (3)	
N1	0.3554 (4)	0.20234 (16)	0.5000	0.0406 (8)	
H1	0.3861	0.2438	0.5000	0.049*	
N2	0.2710 (4)	0.09024 (15)	0.5000	0.0443 (8)	
H2	0.2394	0.0489	0.5000	0.053*	
C1	0.1931 (4)	0.20125 (18)	0.5000	0.0433 (9)	
H1A	0.1563	0.2231	0.3961	0.052*	0.50
H1B	0.1563	0.2231	0.6039	0.052*	0.50
C2	0.1428 (5)	0.1332 (2)	0.5000	0.0537 (12)	
H2A	0.0836	0.1251	0.6039	0.064*	0.50
H2B	0.0836	0.1251	0.3961	0.064*	0.50
C3	0.4100 (3)	0.17034 (16)	0.6617 (5)	0.0535 (8)	
H3A	0.3730	0.1921	0.7657	0.064*	
H3B	0.5158	0.1720	0.6646	0.064*	
C4	0.3595 (4)	0.10141 (15)	0.6615 (5)	0.0541 (8)	
H4A	0.4432	0.0729	0.6626	0.065*	
H4B	0.3013	0.0929	0.7660	0.065*	
O	0.3071 (4)	−0.04203 (13)	0.5000	0.0522 (8)	

### Atomic displacement parameters (Å^2^)

**Table:**

	*U*^11^	*U*^22^	*U*^33^	*U*^12^	*U*^13^	*U*^23^
Sb	0.03290 (16)	0.02973 (15)	0.03718 (16)	−0.00263 (10)	0.000	0.000
Cl1	0.0468 (6)	0.0716 (8)	0.0850 (9)	0.0239 (6)	0.000	0.000
Cl2	0.0379 (5)	0.0543 (6)	0.0492 (6)	0.0080 (4)	0.000	0.000
Cl3	0.0517 (4)	0.0401 (4)	0.0483 (4)	−0.0104 (3)	−0.0131 (3)	0.0003 (3)
Cl4	0.0639 (7)	0.0548 (6)	0.0414 (5)	0.0027 (5)	0.000	0.000
N1	0.0392 (17)	0.0292 (15)	0.053 (2)	−0.0064 (13)	0.000	0.000
N2	0.050 (2)	0.0268 (15)	0.056 (2)	0.0011 (14)	0.000	0.000
C1	0.036 (2)	0.0315 (18)	0.062 (3)	0.0061 (16)	0.000	0.000
C2	0.0314 (19)	0.039 (2)	0.091 (4)	−0.0053 (18)	0.000	0.000
C3	0.0447 (16)	0.0601 (19)	0.0557 (19)	−0.0037 (14)	−0.0143 (15)	−0.0024 (16)
C4	0.0640 (19)	0.0475 (16)	0.0508 (18)	0.0084 (14)	−0.0059 (17)	0.0100 (15)
O	0.0487 (17)	0.0359 (15)	0.072 (2)	−0.0019 (13)	0.000	0.000

### Geometric parameters (Å, º)

**Table:**

Sb—Cl1	2.4520 (12)	N2—H2	0.9100
Sb—Cl3^i^	2.4904 (7)	C1—C2	1.492 (6)
Sb—Cl3	2.4904 (7)	C1—H1A	0.9700
Sb—Cl2	2.8755 (11)	C1—H1B	0.9700
Sb—Cl4	2.9107 (8)	C2—H2A	0.9700
N1—C3	1.481 (4)	C2—H2B	0.9700
N1—C3^i^	1.481 (4)	C3—C4	1.511 (4)
N1—C1	1.487 (5)	C3—H3A	0.9700
N1—H1	0.9100	C3—H3B	0.9700
N2—C2	1.478 (5)	C4—H4A	0.9700
N2—C4^i^	1.485 (4)	C4—H4B	0.9700
N2—C4	1.485 (4)		
			
Cl1—Sb—Cl3^i^	88.39 (3)	N1—C1—H1A	109.9
Cl1—Sb—Cl3	88.39 (3)	C2—C1—H1A	109.9
Cl3^i^—Sb—Cl3	93.37 (3)	N1—C1—H1B	109.9
Cl1—Sb—Cl2	164.64 (4)	C2—C1—H1B	109.9
Cl3^i^—Sb—Cl2	81.11 (2)	H1A—C1—H1B	108.3
Cl3—Sb—Cl2	81.11 (2)	N2—C2—C1	109.4 (3)
Cl4—Sb—Cl3	90.19 (1)	N2—C2—H2A	109.8
Cl4—Sb—Cl3^i^	173.84 (1)	C1—C2—H2A	109.8
Cl4—Sb—Cl1	86.69 (4)	N2—C2—H2B	109.8
Cl4—Sb—Cl2	104.42 (1)	C1—C2—H2B	109.8
C3—N1—C3^i^	111.4 (3)	H2A—C2—H2B	108.2
C3—N1—C1	109.3 (2)	N1—C3—C4	109.0 (3)
C3^i^—N1—C1	109.3 (2)	N1—C3—H3A	109.9
C3—N1—H1	108.9	C4—C3—H3A	109.9
C3^i^—N1—H1	108.9	N1—C3—H3B	109.9
C1—N1—H1	108.9	C4—C3—H3B	109.9
C2—N2—C4^i^	109.8 (2)	H3A—C3—H3B	108.3
C2—N2—C4	109.8 (2)	N2—C4—C3	108.5 (3)
C4^i^—N2—C4	110.8 (4)	N2—C4—H4A	110.0
C2—N2—H2	108.8	C3—C4—H4A	110.0
C4^i^—N2—H2	108.8	N2—C4—H4B	110.0
C4—N2—H2	108.8	C3—C4—H4B	110.0
N1—C1—C2	108.9 (3)	H4A—C4—H4B	108.4

Symmetry code: (i) *x*, *y*, −*z*+1.

### Hydrogen-bond geometry (Å, º)

**Table:**

*D*—H···*A*	*D*—H	H···*A*	*D*···*A*	*D*—H···*A*
N1—H1···Cl2	0.91	2.59	3.340 (4)	140
N1—H1···Cl3	0.91	2.77	3.390 (3)	126
N1—H1···Cl3^i^	0.91	2.77	3.390 (3)	126
N2—H2···O	0.91	2.00	2.780 (4)	143

Symmetry code: (i) *x*, *y*, −*z*+1.
